# Origin of yield stress and mechanical plasticity in biological tissues

**Published:** 2024-09-06

**Authors:** Anh Q Nguyen, Junxiang Huang, Dapeng Bi

**Affiliations:** Department of Physics, Northeastern University, Boston, MA 02115, USA and Center for Theoretical Biological Physics, Northeastern University, Boston, Massachusetts 02215, USA

## Abstract

During development and under normal physiological conditions, biological tissues are continuously subjected to substantial mechanical stresses. In response to large deformations cells in a tissue must undergo multicellular rearrangements in order to maintain integrity and robustness. However, how these events are connected in time and space remains unknown. Here, using computational and theoretical modeling, we studied the mechanical plasticity of epithelial monolayers under large deformations. Our results demonstrate that the jamming-unjamming (solid-fluid) transition in tissues can vary significantly depending on the degree of deformation, implying that tissues are highly unconventional materials. Using analytical modeling, we elucidate the origins of this behavior. We also demonstrate how a tissue accommodates large deformations through a collective series of rearrangements, which behave similarly to avalanches in non-living materials. We find that these ‘tissue avalanches’ are governed by stress redistribution and the spatial distribution of vulnerable spots. Finally, we propose a simple and experimentally accessible framework to predict avalanches and infer tissue mechanical stress based on static images.

## I. INTRODUCTION

During morphogenesis and under normal physiological conditions, biological tissues continuously experience substantial mechanical stresses [1]. Research efforts to understand the remarkable deformability of epithelial tissues employ both experimental and simulation approaches. Experimentally, studies focus on the tissue’s responses to external stresses [2–5], where a stress-driven unjamming transition has been noted [4]. On the simulation front, the cellular Potts and Vertex-based models are utilized to probe tissue rheology [6, 7], uncovering nonlinear elasticity and rheological properties [7, 8]. However, with few exceptions [9], research has predominantly focused on the shear startup regime. This leaves a gap in our understanding of tissue behavior under steady shear and the mechanisms underpinning yield-stress behavior in tissues. Beyond the yield stress, materials typically flow through plastic rearrangements. Similarly, within tissues, mechanical plasticity occurs through cellular rearrangements, enabling the maintenance of integrity and robustness. While there is extensive literature on how individual cells rearrange with their neighbors [10–13], significant gaps remain in understanding how these localized events connect over time and space. Moreover, a major challenge lies in elucidating how these collective interactions lead to mechanical responses at the tissue level. In the context of material plasticity, avalanche-like behavior, prevalent in phenomena ranging from earthquakes to ferromagnets, involves small perturbations triggering significant collective responses [14]. Systems exhibiting these instabilities display self-organized criticality [15] and power law scaling in their observables, indicating the universality class of the process. Proliferation-driven avalanche-like behavior has recently been observed in the Drosophila eye disc, suggesting that avalanches provide a macroscopic mechanism for epithelial tissues to alleviate accumulated proliferative stress [16]. Moreover, shear-induced avalanches have been documented in vertex-based models [8, 9, 17], yet a detailed examination of these avalanches’ growth is still lacking.

In this work, we investigate tissue mechanical plasticity using the Voronoi-based Vertex model under quasi-static shear. Our results demonstrate that the solid-fluid transition point—also referred to as the jamming-unjamming transition in recent literature—does not occur at a singular point but varies depending on the degree of shear deformation the tissue undergoes. Furthermore, challenging traditional definitions, we discover states where tissues possess yield-stress properties but lack a conventional shear modulus. These states exist in a solid-fluid coexistence phase near the jamming-unjamming transition, which we explore through a modified version of the Soft Glassy Rheology (SGR) model to elucidate the origins of these complex states. Our research not only clarifies how tissue manages large deformations through multicellular rearrangements akin to avalanches observed in non-living materials but also connects these phenomena to the tissue-level mechanical responses discussed earlier. These “tissue avalanches” are driven by stress redistribution and the spatial distribution of vulnerable spots, elements that echo the earlier discussions on mechanical responses and rheological properties. By quantifying the spatial and temporal correlations within these rearrangements, we advance the field’s understanding and propose a novel methodological framework capable of predicting collective rearrangements and inferring tissue stress from static snapshots of tissue configuration.

## II. RESULTS

### A. The confluent jamming transition is not unique

To investigate the mechanical behavior of dense epithelial tissues under substantial deformation, we employed a Voronoi-based Vertex model [8, 18]. The cell centers $\left\{r_{i}\right\}$ and their geometric configurations are derived from Voronoi tessellation. The biomechanical interactions are captured through a dimensionless mechanical energy functional [19] expressed as:

(1) $$\varepsilon =\sum_{i=1}^{N}\;[\kappa_{A}{\left(a_{i}-1\right)}^{2}+{\left(p_{i}-p_{0}\right)}^{2}],$$

where $a_{i}$ and $p_{i}$ are the dimensionless area and perimeter of each cell, $\kappa_{A}$ is the rescaled area elasticity, and $p_{0}$ is the *preferred cell shape index* (see Methods). To probe tissue response, we applied quasi-static simple-shear deformation using Lees-Edwards boundary conditions, incrementally increasing strain with the FIRE algorithm to minimize energy (see Methods).

In the unsheared version of the vertex model, it has been demonstrated that the preferred cell shape index $p_{0}$ drives a rigidity transition at $p_{0}=p_{0}^{*}\approx 3.81$, where the linear response shear modulus vanishes [20]. Recent studies have shown that beyond this transition point, in the liquid phase $\left(p_{0}>p_{0}^{*}\right)$, the model can undergo strain-stiffening, indicating a rigidity gain upon strain application. In our quasi-static shearing protocol, we explore beyond the linear response and shear startup regimes into the large deformation limit. In this regime, the tissue exhibits plastic flow primarily through cell-cell rearrangements, or T1 transitions. Here the mean shear stress corresponds to the tissue’s yield stress

(2) $$\sigma_{yield}=\underset{\dot{\gamma }\to 0}{lim}\;\left\langle \sigma_{xy}(\dot{\gamma })\right\rangle .$$

**FIG. 1.**
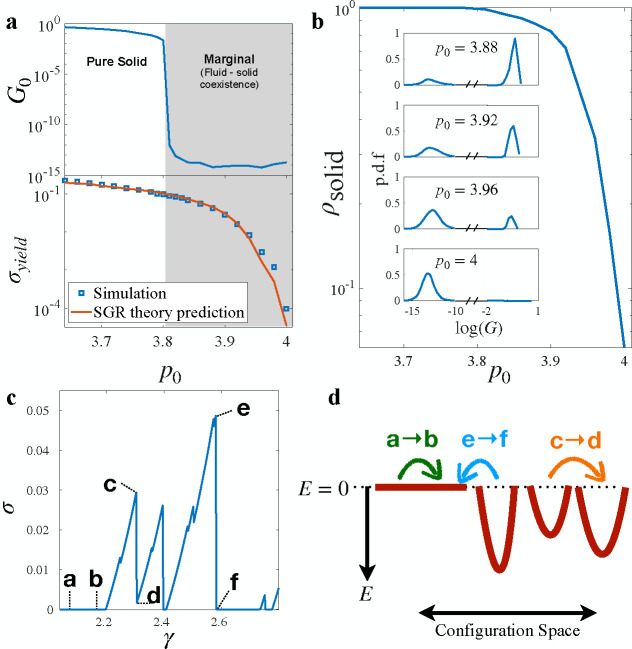
(**a**) Discrepancy between shear start-up and steady-shear regime. Top: The shear modulus of the un-sheared tissue (γ=0). The shear modulus is obtained using linear-response calculation (see Methods). Bottom: The yield stress $\sigma_{yield}$ obtained from the steady-state shear regime of quasistatic simulations is shown together with the yield stress obtained from the SGR model, where the only fitting parameter in the model, the elastic constant of an element was chosen to be k=0.0386. (**b**) The probability of finding the system in solid state as a function of $p_{0}$. Inset: Distribution of tissue stress at different $p_{0}$. (**c**) Stress-strain curve example showing different yielding types: a fluid state yields to another fluid states a→b, a solid state yields to another solid one c→d, and a solid state yields to a fluid state e→f. (**d**) Schematic of the dynamics of elements in the SGR model: The energy landscape of the material consists of traps with different depth E drawn from a distribution ρ(E) that characterizes the structural disorder of the material. Yielding events are captured by transitions from one trap to another. The three types of transition illustrates the transitions observed in simulation

As illustrated in Fig.1a, while the startup shear modulus vanishes at the rigidity transition at $p_{0}\approx 3.81$, signaling a solid-to-fluid transition, the yield stress $\sigma_{yield}$ does not disappear. Instead, it vanishes at a higher cell shape index, $p_{0}\approx 4$. This underscores a drastic difference in tissue responses between the transient shear startup and steady-shear regimes.

Under steady shear and at shape indices higher than the rigidity transition associated with shear startup (i.e., at $p_{0}>p_{0}^{*}$), initially fluid-like systems can intermittently exhibit solid-like behavior before reverting to fluid-like states after yielding (Fig.1c). In this context, states with finite shear modulus are considered solid-like (e.g., states **c, d, f** in Fig.1c), while states that do not resist shear deformation, indicated by having zero shear modulus, are fluid-like (e.g., states **a, b, e** in Fig.1c). This coexistence therefore shows up as a bimodal distribution of the shear modulus, p.d.f(G) shown in Fig. 1b, where the fluid phase is associated with a peak near the numerical noise floor of shear modulus (~ 10^−12^) while the solid phase corresponds to a finite shear modulus.

The shifting behavior in the distributions can be quantified by the fraction of solid states $\rho_{solid}$ shown in Fig.1b. States below the rigidity transition $p_{0}=p_{0}^{*}\approx 3.81$ therefore are always in the solid phase, which we term a **pure solid**. In the range of $p_{0}\in [3.81,\;4]$, $\rho_{solid}$ drops below 1 indicating a solid-fluid coexistence, which we will refer to as **marginal**. For $p_{0}>4$ the tissue remains always in the fluid phase as it cannot build up stresses in response to shear strain. This is also consistent with the yield stress vanishing at $p_{0}≃4.0$. The fact that the material response depends on the application of shear is reminiscent of shear jamming in granular materials, where an state below the isotropic (un-sheared) jamming threshold can be jammed with the application of shear [21–24]. The coexistence of solid and fluid phases also has analogs in dense suspensions near shear jamming [25] and discontinuous shear thickening [9, 26].

### B. Predicting the tissue yield stress using a refined Soft Glassy Rheology model

Given the continuous behavior of yield stress across the pure solid - marginal state transition, we aimed to develop a unified model to deepen our qualitative and predictive understanding the steady-shear regime properties using the Soft Glassy Rheology (SGR) framework [27, 28]. In the SGR model, mesoscopic elements, characterized by elastic constant k and local strain l, are confined within energy traps E, where they accumulate elastic energy as macroscopic strain increases, approaching a yield point either directly or through an activated “hop” driven by mechanical fluctuations from neighboring elements yielding. The material’s dynamics under shear are governed by the probability (E,l,t) , which follows the stochastic differential equation [27, 28]:

(3) $$\frac{\partial }{\partial t}P(E,l,t)=-\dot{\gamma }\frac{\partial P}{\partial l}-\Gamma_{0}e^{\left[E-kl^{2}/2\right]/x}P+\Gamma (t)\rho (E)\delta (l).$$

Here the first term in Eq. 3 describes the motion of the elements caused by global shear rate $\dot{\gamma }$. The second term captures activated hopping from a trap of depth $E-kl^{2}/2$ (corresponding to the distance to yielding). x represents the mechanical noise in the system akin to temperature. The final term illustrates the transition to new states, with (E,l=0) following yield, selected from a quenched random distribution ρ(E).

In the SGR model, the choice of ρ(E)’s functional form critically influences material behavior [28]. Direct measurement of energy barrier distributions is challenging, leading prior studies to adopt generic or ad hoc assumptions for ρ(E), such as exponential distributions [29–31]. In this work, we introduce a novel approach based on distinct mesoscopic tissue phases observed: (1) fluid elements with zero yielding energy (E=0) and (2) solid elements with finite yielding energy (E>0). Consequently, we propose a refined ρ(E):

(4) $$\rho (E)=f_{0}\delta (E)+\left(1-f_{0}\right)\frac{E^{\kappa -1}e^{-E/x_{0}}}{\Gamma (\kappa )x_{0}^{\kappa }}.$$

Here, $f_{0}$ denotes the probability of an element transitioning to a state with E=0, and $1-f_{0}$ corresponds to transitions into states with energy sampled from a k-gamma distribution, parameterized by mean $x_{0}$ and shape factor κ. This is based on the previous observation that the energy barriers to the T1 transition follow a k-gamma distribution [20, 32] with κ≈2. Together,Eqs. 3 and 4 describe three potential transitions in the energy landscape, depicted in Fig. 1d: (1) fluid to fluid a→b, (2) solid to solid c→d, and (3) solid to fluid e→f.

We next examine the steady state behavior of Eq. 3 in the quasi-static limit $\left(\dot{\gamma }\to 0\right)$, with details shown in the Appendix. The behavior is governed by three parameters: the dimensionless ratio of mechanical noise to mean yielding energy $\chi =x/x_{0}$, the probability $f_{0}$ of transitioning to a fluid state, and the elastic constant k of solid elements.

An important aspect of the SGR model is that the fluctuations that drive element rearrangements come from the mechanical noise due to other surrounding rearrangement events in the system. These fluctuations are analogous to the energies released during yielding events observed in our simulations. To correlate this mechanical noise with our empirical data, we introduce the following relationship:

(5) $$\chi =\frac{x}{x_{0}}\propto \frac{\left\langle \Delta E\right\rangle }{\left\langle E\right\rangle }.$$

Here, ⟨ΔE⟩ represents the average energy dissipated during yielding events, while ⟨E⟩ denotes the average energy of cells in their solid state. Next, by analyzing the steady-state solution of Eq. 3. we determine the probability that an element is in the solid phase as a function of $f_{0}$ (details in SI, Eq. S.12). This corresponds precisely to $\rho_{solid}$ in our simulations (Fig. 1b). Finally, we treat the elastic constant k of the elements as a constant, independent of the shape index $p_{0}$. Given that both ⟨ΔE⟩/⟨E⟩ and $\rho_{solid}$ depend on $p_{0}$, the yield stress predicted by the SGR model (detailed in the SI) effectively varies only with $p_{0}$. This approach contrasts with previous studies that employed the SGR model [29–31, 33], where $\chi =x/x_{0}$ was often used as a fitting parameter. In our research, we derive χ directly from simulation data, enhancing the predictive accuracy of our theoretical results and distinguishing our use of the SGR model as predictive rather than merely descriptive. In Fig. 1a, we plot the SGR-predicted yield stress as a function of $p_{0}$. This demonstrates that the SGR model accurately predicts the yield stress vanishing point and its dependence on the cell shape index $\sigma_{yield}\left(p_{0}\right)$.

**FIG. 2.**
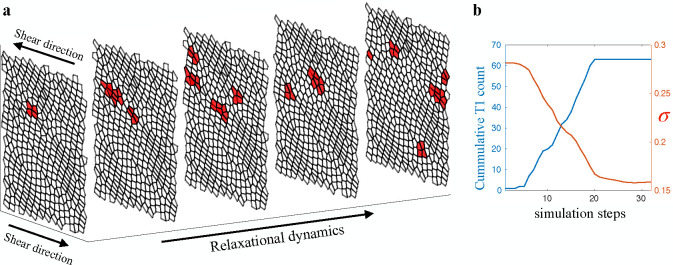
Spatiotemporal map of plastic events in tissue. (**a**) Space-time map of plastics rearrangement in the form of T1 transition during an avalanche. Cells that participated in T1 transitions are labeled in red. The example avalanche was selected from a system at $p_{0}=3.72$. (**b**) Number of accumulative plastic rearrangement and tissue shear stress as the avalanche progresses.

The dual-state SGR model identifies two primary mechanisms responsible for the yield stress transition: (**1**) As $p_{0}$ increases, states with zero yielding energy barriers become more prevalent, leading to frequent yielding under deformation. This behavior is depicted by transitions such as a→b and e→f in Fig. 1d; **(2)** Concurrently, mechanical noise from stress redistributions approaches the scale of the yielding energy barriers, enhancing the likelihood of solid-solid transitions through activated processes induced by neighboring rearrangements, as illustrated by c→d and e→f.

### C. Dynamics of tissue plasticity

So far, we know that for a system in the coexistence phase to transitions from solid to fluid, a collective rearrangement event is required to significantly remodel the configuration. However, the mechanism that govern the occurrences of these events and the evolution of the system during the events is still not fully understood. To explore the yielding behavior of biological tissues, it is essential to describe the dynamics of plastic events during avalanches. In this study, we examine the plasticity dynamics by investigating the spatiotemporal evolution of the plastic rearrangements created by other rearrangements during an avalanche[34, 35]. Fig.2a displays a space-time plot of the occurrences of T1 transitions during an avalanche, with the cells labeled in red indicating participation in the T1 transitions.

As depicted in Fig.2a, an avalanche involving numerous plastic rearrangements could originate from a single T1 transition, which we refer to as the initial trigger. Starting from the initial trigger, the stress redistribution from each event can also stimulate surrounding cells to become unstable and undergo T1 transitions. These vulnerable cells have been referred to as soft spots [36, 37] or STZs [38, 39]. This cascade of cellular rearrangements can therefore lead to an avalanche, which continues until the population of soft spots are sufficiently depleted. In Fig.2b, we show the number of accumulated T1 transitions and the tissue shear stress during a typical avalanche. The stress relaxation due to an avalanche therefore is the origin of the discontinuous yielding of stress during quasistatic shear. In Fig.2a, the location of rearrangments over time suggests that there is a preferred direction for the avalanche to propagate. In order to quantify this and to establish a causal relationship in time, we define a two-point, two-time correlation function:

(6) $$\phi \left(r,\Delta t\right)=\left\langle P\left(r_{0},t_{0}\right)P\left(r_{0}+r,t_{0}+\Delta t\right\rangle \right.,$$

where P(r,t) is a binary field, representing the occurrence of a T1 transition (1 if a T1 transition at occurs at r and time t and 0 otherwise). ⟨…⟩ represents spatial and ensemble averaging. With this definition, ϕ is therefore the conditional probability of observing a T1 transition at location $r_{0}+r$ and time $t_{0}+\Delta t$, given that a transition has already occurred at $\left(r_{0},t_{0}\right)$.

**FIG. 3.**
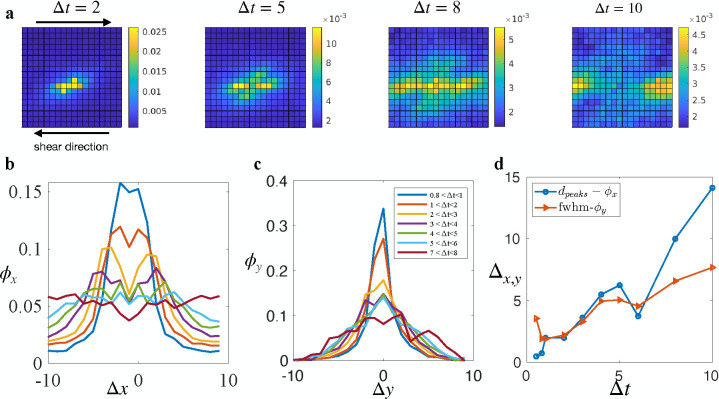
Propagation of plastic events. (**a**) Evolution of the probability field ϕ at $p_{0}=3.72$. Bright regions indicate a high probability of finding another rearrangement in the region relative to the causal rearrangement. (**b**) Spatial distribution of the correlation field $\phi_{x}$ as a function of the relative horizontal position Δx. The distribution has a diffusing bimodal shape, indicating a convection process alongside diffusion. (**c**) Spatial distribution of the correlation field $\phi_{y}$ as a function of the relative vertical position Δy. The distribution is bell-shaped, with the width of the bell increasing as time progresses, indicating a pure diffusion process. (**d**) The separation of the peaks in $\phi_{x}$ and the Full Width at Half Maximum (FWHM) of $\phi_{y}$ as functions of the time lag Δt.

In Fig 3a, we illustrate the evolution of the field ϕ as Δt increases. The field ϕ behaves like a wave that propagates away from the causal rearrangement and prefers to propagate in the x-direction, coinciding with the direction of the external shear force. The evolution of the field ϕ reflects the interplay between the stress redistribution from a plastic rearrangement and the population of soft spots which could rearrange under the effect of the strain field.

We first focus on the angular dependence of ϕ, which shows an anisotropic four-fold pattern. This anisotropy is consistent with the stress redistribution field due to a rearrangement in an elastic medium as predicted by elastoplastic models [17, 40]. However, it differs from the isotropic probability field observed in ductile, soft disk systems [41]. This contradiction likely arises from the difference in shape anisotropy between soft disks and cells in our system. While soft disk systems exhibit minimal particle shape anisotropy, cells in our system can sustain large deformations and have highly anisotropic shapes. Consequently, deviatoric strain triggers rearrangements in soft disk systems, whereas simple shear strain is responsible for triggering rearrangements in our system.

To better understand how the rearrangement probability field propagates, we looked at $\phi_{x}=\frac{\phi (x,y=0)}{\sum_{x}\;\phi (x,y=0)}$ and $\phi_{y}=\frac{\phi (x=0,y)}{\sum_{y}\;\phi (x=0,y)}$ separately. $\phi_{x}$, as observed in Fig.3b, is a bimodal distribution that evolves in time such that the distance between the two peaks $d_{peaks}$ increases as time goes. The diffusing bimodal distribution suggests that the propagation is a combination of convection and diffusion, and the drift velocity could be captured by the rate at which the peaks’ separation increases. Conversely, $\phi_{y}$ is a bell-like shape distribution that gets broader as time progresses (Fig.3c), indicating that the propagation in the y-direction is similar to a purely diffusion process with the diffusivity can be captured by the evolution of the FWHM. Fig.3d shows that $d_{peaks}$ increases faster than the FWHM-$\phi_{y}$. Since the rearrangement probability field is the result of the shear stress redistribution and the population of soft spots, the propagating mechanism of the field also should agree with the propagation of the stress redistribution.

The phenomenon that the shear stress redistribution tends to propagate in the direction of shear has also been observed in particle-based systems governed by inverse-power-law pairwise potentials [35]. Remarkably, this behavior bears a striking resemblance to the propagation of elastic waves. In the theory of elasticity, longitudinal waves, characterized by displacement in the direction of propagation, outpace transverse waves [42]. Moreover, longitudinal elastic waves involve changes in local density[42], akin to the x-propagating excitation wave’s modulation of local density via T1 transitions. Conversely, transverse elastic waves do not induce density changes, resembling the infrequent involvement of T1 transitions in y-propagating excitation waves. Notably, the mechanism driving stress redistribution to preferentially propagate in the shear direction appears universal, independent of $p_{0}$. However, since $p_{0}$ governs the elastic response in our system, with higher values corresponding to a less elastic state, there is a negative correlation between the stress redistribution wave’s speed and $p_{0}$.

### D. Statistics of tissue avalanches

**FIG. 4.**
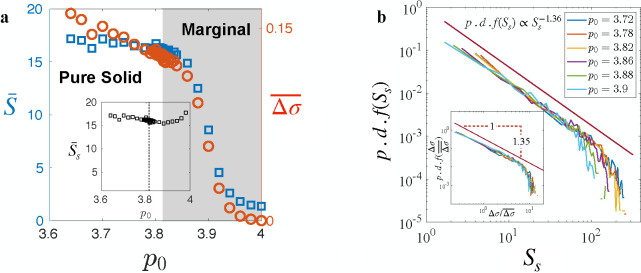
Avalanches statistics. (**a**) Dependence of average yielding size $\overset{‾}{S}$ and average stress relaxed $\bar{\Delta \sigma }$ on $p_{0}$. Inset: Dependence of average avalanches size ${\overset{‾}{S}}_{s}$ on $p_{0}$. (**b**) Distribution of avalanche size follows a power-law distribution. Inset: Distribution of scaled average stress relaxed by avalanches also follows the same power law

In addition to the universal propagation mechanism, we wondered if the statistics of tissue yielding events also exhibit universality. In Fig.4a, both the average yielding size $\overset{‾}{S}$, denoting the total number of T1 transitions after a yielding event, and the average stress drop, representing the amount of stress relaxed by the event, exhibit the same dependence on $p_{0}$. In the solid regime, while the stress decreases with increasing $p_{0}$, the average yielding size shows minimal variation. This trend of $\overset{‾}{S}$ versus $p_{0}$ is akin to that observed in Fig.1b for the proportion of the solid state.

However, in the marginal phase, there are different types of yielding events as discussed previously (Fig.1c). In the yielding events that occur while the system is fluid-like, illustrated by the a→b transition in Fig.1C (Type **I**), the tissue lacks rigidity and therefore is unable to transmit stress to initiate a cascade of rearrangements. Conversely, yielding events following a solid state, illustrated by c→d and e→f transitions (Type **II**), tend to be cascading as the rigid tissue is capable of propagating the stress redistribution. It is this latter type that we refer to as tissue avalanches from now on. Since the avalanches growing mechanism is universal, we expect their statistics to also be independent of $p_{0}$.

Excluding yielding events of type **I** from the analysis and specifically analyzing only the avalanches, we indeed find that the average avalanche size $\bar{S_{s}}$ does not vary significantly with $p_{0}$ (Fig.4a inset), suggesting universal avalanches statistics. To rigorously assess this universality, we examine the distribution of avalanche sizes across various $p_{0}$ values (see Fig.4b). Strikingly, we observe a consistent power-law distribution, reminiscent of the Gutenberg–Richter (GR) law observed in earthquakes [43, 44], with an exponent τ=−1.36, which agrees with the analogous exponent observed in overdamped elastoplastic models under shear [45, 46] and in vertex model on spherical surface[47]. This shared characteristic suggests a parallel between biological and seismic avalanches and supports the argument that the vertex model and epithelial tissues belong to the universality class of plastic amorphous systems.

Furthermore, we find that the same power law applies to the distribution of average stress drops during avalanches when scaled by the average (Fig.4b inset). This collapse after rescaling implies that the stress relaxation mechanism via avalanches is independent of the shape index $p_{0}$, and $p_{0}$ only affects the average stress relaxed by governing tissue overall stiffness. Moreover, the similarity in the stress drop distribution and avalanches size distribution indicates that each plastic rearrangement, on average, releases a similar amount of stress that depends only on $p_{0}$. The convergence of these distributions suggests that the growth of avalanches remains unaffected by changes in $p_{0}$, providing additional evidence for the universal propagation mechanism discussed earlier.

### E. Predicting tissue avalanches based on static structural information

While the first cells to undergo a T1 transition triggers the avalanche, in order for the avalanche to grow, it is necessary to have soft spots in the system that are susceptible to undergo T1s. In the framework of the elastoplastic model, it has been established that the distance to yield x, which represents the additional stress required to trigger a yielding event, follows a power-law distribution, $p(x)\propto x^{\theta }$ [48–50]. The exponent θ has been suggested as a measure of the system’s instability, with a higher value indicating a more stable state.

In the vertex-based model family, it has been proposed that the distance to yield, x, exhibits a linear relationship with the length of cell edges, and that the distribution of edge lengths should follow the same power-law behavior as ρ(x) [17]. While this argument establishes a connection between system configuration and instability, the efficacy of using the distribution of short edges to describe instability remains uncertain.

**FIG. 5.**
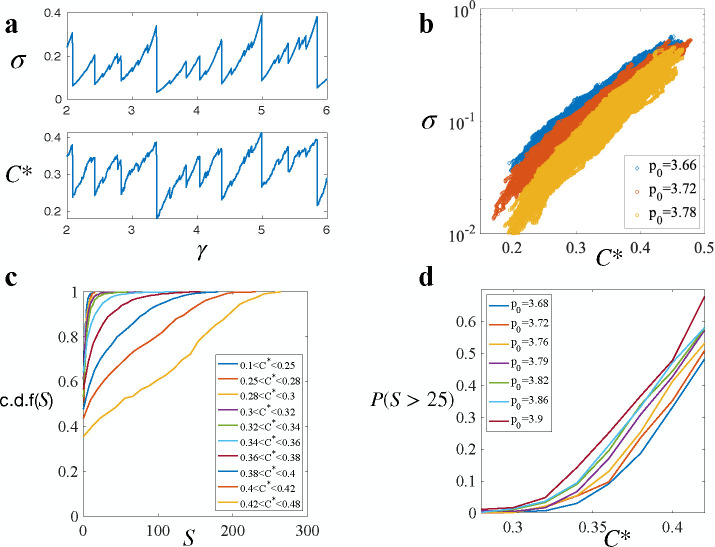
Predicting avalanches. (**a**)An example of the evolution of tissue shear stress $\sigma_{xy}$ and $C^{*}$ as strain increases. **b**) Scatter plot of shear stress and $C^{*}$ at different $p_{0}$ in the solid regime. **c**) c.d.f(S) at different $C^{*}$ level at $p_{0}=3.79$. **d**) Probability of having avalanches of size greater than 25 at different $C^{*}$

To address this ambiguity, we investigate this concept within our Voronoi model and observed an intriguing correlation between the exponent θ and system instability (see S.I and Fig.S1). However, θ is not a reliable metric because it is derived from a power-law fit that heavily depends on the range of fitting [17]. In practice, the cumulative distribution function (c.d.f) of L exhibits power-law behavior only within a specific range, which varies from sample to sample. Therefore, we propose using $c.d.f(L^{*})$ as the parameter of instability to avoid the uncertainties and biases associated with fitting. To systematically determine a critical edge length $L^{*}$, we analyzed the evolution of the edge length distribution, c.d.f(L), at various L values and compared it with the evolution of tissue shear stress $\sigma_{xy}$ (Fig.5a). We observed a critical $L^{*}$ at which $c.d.f(L^{*})$, denoted by $C^{*}$ from now, exhibited an exceptionally strong correlation with the stress $\sigma_{xy}$, with a correlation coefficient exceeding 0.9 (Fig.5a). Interestingly, we found that $L^{*}=0.43$ (in units of the average cell diameter) remains consistent across different $p_{0}$ values.

Given the high correlation between $C^{*}$ and σ, and the fact that cell edge lengths can be directly extracted from imaging, $C^{*}$ could serve as a non-invasive metric to infer tissue-level stress. Plotting $C^{*}$ against $\sigma_{xy}$ for $p_{0}$ in the plastic regime reveals a clear exponential relationship, as shown in Fig.5b. However, due to the limited range of $C^{*}$, it is challenging to distinguish between an exponential and a power-law relationship. Therefore, we tested both the exponential model $\left(\sigma_{xy}\propto e^{\alpha C}\right)$ and the power-law model $\left(\sigma_{xy}\propto C^{\beta }\right)$. Both models demonstrated a good representation of tissue shear stress (Fig.S4 a, b), with the exponential model exhibiting a slightly lower mean-squared error (MSE) compared to the power-law model (Fig.S4e).

Using $C^{*}$ as the parameter of instability, with higher $C^{*}$ corresponding to a more unstable state, we observed a relationship between instability and avalanche size. By selecting only the failure states, we computed $C^{*}$ for these states and then grouped the avalanche sizes based on $C^{*}$ at the point of failure (Fig.5c). At low $C^{*}$, if the tissue yields, the size of the yielding event is likely to be small, indicated by a rapid increase to 1 in the cumulative distribution function (c.d.f.) of S. As $C^{*}$ increases, the likelihood of larger yield events grows. The probability of observing an avalanche of size 25 or greater is summarized in Fig.5d.

While $C^{*}$ can provide predictions about avalanche size, it cannot determine when an avalanche will occur. Therefore, we require another tool to forecast yielding events. In amorphous solids, the locations of plastic rearrangements during an avalanche largely depend on the material’s structural configuration, with areas more likely to experience plastic events called soft spots. Various frameworks have been proposed to link structure and plasticity, such as the Shear Transformation Zone theory [37–39, 51, 52] and lattice-based models. The most promising theoretical approach for predicting the locations of soft spots involves identifying these areas based on soft vibrational modes [36, 53, 54]. As a system approaches a plastic rearrangement, at least one normal mode is supposed to approach zero frequency [36]. However, the vibrational mode analysis is not applicable to the vertex-based model family due to the cuspiness of the energy landscape [20, 32]. In such systems, the energy cusp at a plastic event prevents the corresponding low-frequency mode from vanishing as it would in systems with smooth, analytic energy landscape. As shown in Fig.S2, in our system, no low-frequency mode approaches zero frequency except at the onset of the plastic event. Hence, an alternative approach is necessary.

In our model, the deformation of edges immediately following shear strain is deterministic. Through simple geometry, we deduce the existence of a range of orientations wherein edges are prone to shortening upon shearing, rendering them more vulnerable. In the vertex model, under the condition $\dot{\gamma }\ll 1$, the change in edge length δl due to shear is approximated as $\delta l\approx \dot{\gamma }l\;sin(2\phi )$, where l and ϕ represent the edge length and orientation, respectively. Consequently, in the vertex model, the most vulnerable orientation is $\frac{3\pi }{4}$. If an edge is sufficiently weak (or short) and happens to align with this susceptible orientation, it may yield under the influence of shearing, potentially triggering further rearrangements in its vicinity. We refer to these susceptible edges as triggers. Since triggers are local elements, their presence is not captured by the cumulative distribution function of edge lengths, c.d.f(L), thereby explaining why $C^{*}$ alone cannot predict imminent system failure. In summary, the presence of a trigger is the necessary condition, while a high $C^{*}$ in the current state is the sufficient condition for a large avalanche in a tissue monolayer.

## III. DISCUSSION

We studied the response of tissue monolayers to external quasi-static shear stress in the long-term steady-shear regime. For a tissue monolayer initially in a fluid state, it behaves like a yield-stress material in the shear buildup regime but eventually enters a marginalphase in the long-term steady shear regime. Incorporating the coexistence phase into to the SGR model, we elucidated the discrepancy between the rigidity transition and the yield stress transition observed in the Vertex model under simple shear.

Besides the yield stress, tissue plasticity is also reflected in avalanches of plastic rearrangement. By studying the dynamics of tissue avalanches, we observed a universal propagation mechanism of plastic events that is independent of the shape index $p_{0}$ and has two preferred directions, with the direction of the external shear being the one with faster propagation. The rearrangement probability ϕ studied here is closely related to, but not identical with, the stress redistribution predicted by elastoplastic models [17, 40] or the strain field due to rearrangement [41]. Instead, ϕ captured the interplay between stress redistribution and spatial distribution of weak spots. Since most edges that participated in T1 transition in our system orient at −π/4 with respect to the horizontal (Fig.S3), the positive shear stress is expected to symmetrically redistribute along the horizontal and vertical directions [17]. Although ϕ does propagate mainly in the horizontal and vertical directions, it does not possess the vertical-horizontal symmetry seen in stress redistribution or in the strain field. This difference arises from highly heterogeneity and anisotropic in the spatial distribution of weak spots.

The universality of tissue avalanches is not only reflected in the propagation of plastic events but also captured by a power-law distribution of avalanche sizes, with an exponent τ=−1.36, strengthening the argument that epithelial tissues behave like plastic amorphous materials. We also propose a metric to not only predict tissue avalanches but also infer tissue stress in highly anisotropic systems based on an instantaneous static snapshot. In finite size system, the cut off avalanches size $S_{c}$ depends on the system size N as $S_{c}\propto N^{d_{f}/d}$, where $d_{f}$ is avalanches fractal dimension and d is the dimension of the system [49]. Future possible work can be performed with different system size to obtain the avalanches fractal dimension $d_{f}$ and further understand the finite size effect on avalanches.

We also propose a metric to not only infer tissue stress based on an instantaneous static snapshot but also predict tissue avalanches. Quantification of tissue-level force and stress is necessary to understand the physics of many biological processes. However, direct measurement of stress in vivo is considerably challenging[55–57]. Compared to other non-invasive method to estimate tissue stress such as Bayesian Force Inference [58] and Variational Method for Image-Based Inference [59], our approach using $C^{*}$ offers a simple and fast method to estimate tissue stress. The cost for this convenience is that our approach cannot provide a spatial distribution of stress. The strong agreement between $\sigma_{xy}$ and $C^{*}$ is noteworthy, especially since $C^{*}$ does not incorporate information about edge orientation, which directly affects stress. In an isotropic system, edge length alone is insufficient to infer stress. However, in a system undergoing large deformation and thus highly anisotropic, the influence of edge orientation diminishes, making edge length alone sufficient for stress inference. The impact of shape anisotropy is evident during the buildup phase or when the system is in a fluid state. In these scenarios, edges may have negligible tension, making edge tension independent of edge length. Consequently, it is possible that systems with similar $\sigma_{xy}$ values could exhibit significantly different stress levels.

The impact of triggers on avalanches goes beyond simply initiating them; we observed a significant dependence of avalanche size on the trigger location. By manually shrinking vanishing edges at various locations within the same configuration, we noted that the size of the resulting avalanches varied markedly. This indicates that the location of the initial excitation has a profound influence on the final size of the avalanche. A promising future research direction could involve developing a theoretical framework that moves beyond the mean field approach to more accurately capture the spatial heterogeneity in the tissue.

## MATERIALS AND METHOD

### Simulation Model

To numerically study the behavior of dense epithelial tissues under large deformation, we use a Voronoi-based version [18] of the Vertex Model [60], where the degrees of freedom are the set of cell centers denoted as $\left\{r_{i}\right\}$ and the geometric configurations of cells are derived from their respective Voronoi tessellation. The biomechanics governing interactions both within and between cells can be effectively represented at a coarse-grained level [19, 61], expressed in terms of a mechanical energy functional associated with cell shapes, given by:

(7) $$E=\sum_{i=1}^{N}\;\left[K_{A}{\left(A_{i}-A_{0}\right)}^{2}+K_{P}{\left(P_{i}-P_{0}\right)}^{2}\right],$$

where $A_{i}$ and $P_{i}$ represent the area and perimeter of the i-th cell, respectively. The parameters $K_{A}$ and $K_{P}$ denote the area and perimeter moduli, respectively. The values $A_{0}$ and $P_{0}$ correspond to the preferred area and perimeter values, with $A_{0}$ specifically set to the average area per cell $\overset{‾}{A}$. Without the loss of generality, we choose $K_{P}A_{0}$ as the energy unit and $\sqrt{A_{0}}$ as the length unit. This leads to the dimensionless form of the energy

(8) $$\varepsilon =\sum_{i=1}^{N}\;\kappa_{A}{\left(a_{i}-1\right)}^{2}+{\left(p_{i}-p_{0}\right)}^{2},$$

where $\kappa_{A}=K_{A}A_{0}/K_{P}$ represents the rescaled area elasticity, governing the cell area stiffness relative to the perimeter stiffness and $p_{0}=P_{0}/\sqrt{A_{0}}$ the cell shape index. To investigate the mechanical response of the tissue, we apply simple-shear deformation to the simulated tissues utilizing Lees-Edwards boundary conditions [62]. Initially, strain-free configurations (γ=0) are generated with randomly distributed cell centers. The FIRE algorithm [63] is subsequently employed to minimize the energy functional in accordance with Eq. 8. Strain is then incrementally applied in steps of $\Delta \gamma =2\times 10^{-3}$ until reaching a maximum value of $\gamma_{max}=6$. Alongside the modification of periodic boundary conditions to account for the strain, an affine displacement field $\Delta r_{i}=\Delta \gamma \;y_{i}\;\overset{ˆ}{x}$ is applied to the cell centers. Following each increment of strain, the FIRE algorithm is again utilized to minimize the energy functional (Eq,8 until the residual forces acting on cell centers fall below 10^−14^, so that all resultant tissue states are metastable. This procedural approach effectively corresponds to investigating the system within the athermal quasi-static limit $\left(\dot{\gamma }\to 0\right)$. The tissues under examination encompass cell populations N=400, accompanied by cell shape indices $p_{0}$ varying between 3.7 and 4, and a total of 84 random initial samples were simulated at each set of parameter values.

We calculate the tension, denoted as $T_{ij}$, acting along an edge $l_{ij}$ shared by cells i and j using the equation [58, 64, 65]

(9) $$T_{ij}=\frac{\partial \varepsilon }{\partial l_{ij}}=2\left[\left(p_{i}-p_{0}\right)+\left(p_{j}-p_{0}\right)\right]{\overset{ˆ}{l}}_{ij},$$

where ${\overset{ˆ}{l}}_{ij}$ represents the unit vector along $l_{ij}$. Furthermore, the global tissue shear stress σ can be obtained by

(10) $$\sigma =\sigma_{xy}\equiv \frac{1}{N}\sum_{i<j}\;T_{ij}^{x}l_{ij}^{y},$$

where $T_{ij}^{x}$ denotes the x-component of $T_{ij}$ and $l_{ij}^{y}$ stands for the y-component of $l_{ij}$.

## Supplementary Material

Supplement 1
