# Supplementary material for: Origin of yield stress and mechanical plasticity in biological tissues
Source: ArXiv. 2024 Sep 6:arXiv:2409.04383v1. Preprint. [Version 1] (PMC11398538)
Supplement: Supplementary file 1 [file NIHPP2409.04383v1-supplement-1.pdf]

APPENDIX

Computing tissue level mechanical linear response

The tissue level mechanical response was quantified by the shear modulus G. We computed G using Born-Huang formulation in the limit of infinitesimal affine strain γ [66, 67]:

$$G = \frac{1}{A_{total}}\left(\frac{\partial^2 E}{\partial \gamma^2} - \Xi_{i\alpha} H^{-1}_{i\alpha j\beta} \Xi_{j\beta}\right), \tag{S.1}$$

where the Roman indexes i, j label cells and Greek indexes α, β denote Cartesian components. $\Xi_{i\alpha}$ is the derivative of the force on cell ith with respect to the strain γ:

$$\Xi_{i\alpha} = \frac{\partial^2 E}{\partial r_{i\alpha} \partial \gamma} \tag{S.2}$$

H is the Hessian matrix given by the second derivative of the tissue energy E with respect to position cells position:

$$H_{i\alpha j\beta} = \frac{\partial^2 E}{\partial r_{i\alpha} \partial r_{j\beta}} \tag{S.3}$$

Steady state solution and asymptotic behavior in dual-state SGR

In the Fokker-Planck equation of motion Eqn. 3, the total yielding rate $\Gamma(t)$ is given by [28]:

$$\Gamma(t) = \Gamma_0 \int dE\, dl\, P(E,l,t) \exp\left[-\frac{E - kl^2/2}{x}\right]. \tag{S.4}$$

Since we are interested in the long-term steady shear, we look for a steady state solution to Eqn. 3. In steady state, Eqn. 3 becomes an ODE with respect to l:

$$\frac{\partial P}{\partial l} + \frac{\Gamma_0}{\dot\gamma} \exp\left[-\frac{E - kl^2/2}{x}\right] P = \frac{\Gamma}{\dot\gamma} \rho(E)\delta(l)$$

The steady-state solution is [28]:

$$P(E,l) = \frac{\Gamma}{\dot\gamma} \rho(E) \exp(-ze^{-E/x}) \tag{S.5}$$

Where $z(l)$ is:

$$z(l) = \frac{\Gamma_0}{\dot\gamma} \int_0^l dl' e^{kl'^2/2x} \tag{S.6}$$

In steady state, the total yielding rate Γ is just a constant and can be found by normalizing $P(E,l)$, giving the steady state solution for $P(E,l)$ of the form:

$$P(E,l) = \frac{\rho(E) \exp(-ze^{-E/x})}{\int_0^\infty dl\, G_\rho(z)}$$

Where G_ρ is:

$$G_\rho(z) = \int_0^\infty dE\, \rho(E) \exp(-ze^{-E/x})$$

G_ρ can be separated into two parts. The first part, denoted by G_δ, comes from the contribution of the zero energy traps (the Dirac-delta function in $\rho(E)$). G_Γ denotes the second part coming from the non-zero energy traps (the Gamma function in $\rho(E)$):

$$G_\delta(z) = \int dE\, \delta(E) \exp(-ze^{-E/x}) = \exp(-z)$$

$$G_\Gamma(z) = \int dE\, \frac{E^{\kappa-1} e^{-E/x_0}}{\Gamma(\kappa) x_0^\kappa} \exp(-ze^{-E/x})$$

$$G_\rho(z) = f_0 G_\delta + (1 - f_0) G_\Gamma$$

The steady-state solution in the long-time limit can be studied more conveniently using the following auxiliary functions:

$$I_{0\delta} = \int dl\, G_\delta$$
$$I_{1\delta} = k_\delta \int dl\, l G_\delta \approx 0$$
$$I_{0\Gamma} = \int dl\, G_\Gamma$$
$$I_{1\Gamma} = k \int dl\, l G_\Gamma$$

Since our main focus is the rheological response of the system, reflected in the macroscopic stress, we compute the system stress by ensemble averaging the local stress [28]:

$$\sigma = \langle kl \rangle = \int\int dE dl\, klP(E,l) \tag{S.7}$$

Using the auxiliary functions and the steady-state solution, the stress is therefore given by:

$$\sigma = \frac{(1-f_0)I_{1\Gamma}}{f_0 I_{0\delta} + (1-f_0)I_{0\Gamma}} \tag{S.8}$$

For an element with zero yielding energy, the strain of the element is of order $\frac{\dot{\gamma}}{\Gamma_0}$. Therefore, in the low strain-rate limit, the strain of the element with zero yield energy is typically small so G_δ can be approximated in this limit as:

$$G_\delta = \exp(-l\Gamma_0/\dot{\gamma})$$

In the case of $\kappa = 2$ and let $\chi = \frac{x}{x_0} < 2$, using the substitution $u = e^{-E/x}$ and integration by part, $G_\Gamma(z)$ can be integrated as follow:

$$\begin{aligned} G_\Gamma(z) &= \int_1^0 \chi^2 \ln(u) u^{\chi-1} \exp(-zu) du \\ &= \frac{\chi^2(\chi-1)!}{z^\chi} \sum_{n=1}^{\infty} \frac{(-z)^n}{nn!} \end{aligned}$$

The series $\sum_{n=1}^{\infty} \frac{(-z)^n}{nn!}$ converges by alternating series test and therefore $G_\Gamma(z)$ scales as $z^{-\chi}$. We then obtain the following scaling relations:

$$I_{0\delta} \approx \dot{\gamma}/\Gamma_0 \tag{S.9}$$
$$I_{0\Gamma} \approx C(\chi)\dot{\gamma}^\chi \tag{S.10}$$
$$I_{1\Gamma} \approx D(\chi)\dot{\gamma}^\chi \tag{S.11}$$

Using, the steady state solution, the proportion of time that elements spends in the zero yielding energy traps (fluid state) P can be expressed in terms of f_0:

$$P = \frac{f_0 I_{0\delta}}{f_0 I_{0\delta} + (1-f_0)I_{0\Gamma}} \tag{S.12}$$

Combining Eq. S.8 and Eq. S.12, the yield stress can be calculated as:

$$\sigma_y(x) = \frac{(1-f_0)I_{1\Gamma}}{f_0 I_{0\delta} + (1-f_0)I_{0\Gamma}} = (1-P)\frac{I_{1\Gamma}}{I_{0\Gamma}} = (1-P)\sigma_0 \tag{S.13}$$

Where σ_0 is the yield stress that arises solely from elements in a solid state, which is finite based on the scaling relation S.10 and S.11.

AN EXPONENT OF INSTABILITY

We extract the instability exponent, θ, from the cumulative distribution function (c.d.f) of edge lengths, denoted as c.d.f(L), by fitting a power law to c.d.f(L) in the interval $0.05 < L < 0.5$. This interval specifically represents the short edges that are capable of undergoing T1 transitions.

We focused on tissues exclusively in the solid regime (shape index p_0 less than 3.81) and integrated this understanding with the dual-state coexistence proportion to extend the analysis to higher shape indices. For purely solid tissues, as stress builds up, the instability exponent θ gradually decreases. Conversely, when stress is relieved through avalanches, θ experiences a sharp increase (S1**a**), indicating that a significant number of soft spots are relaxed, making the system considerably more stable.

Furthermore, the θ exponent is correlated with avalanche properties, as evidenced by its relationship with the average avalanche size ($\bar{S}$) and the probability of avalanche occurrence. Systems with a lower θ exponent, indicating greater instability, tend to experience larger avalanches on average (Figure S1**b**) and are more prone to yielding (Figure S1**b** inset).

NORMAL MODES ARE UNABLE TO IDENTIFY THE SOFT SPOTS IN THE VERTEX-BASED MODEL

In amorphous solids, localization plays a crucial role in understanding the rheology of the material. An example of localization is shear transformation zone (STZ) [37–39, 51, 52], localized regions in which sudden and irreversible rearrangements occur when the material is subjected to shear. These STZ, also referred to as weak spots, can interact and lead to avalanches of irreversible plastic events, making the identification of these weak spots in disordered systems a crucial and challenging task. Research has indicated that the local yield stress could serve as a reliable predictor for these weak spots [37]. However, locally probing the system is impractical and does not align with our objective of making predictions based solely on current and historical snapshots. Another approach to this task involves analyzing the normal mode of the system near failure. Studies on systems of harmonic repulsive particles have demonstrated that low-frequency modes typically correspond to low energy barriers [68], making them dominant modes during a plastic event [68–70]. Furthermore, in systems with explicit separation dependence potential (such as Hertzian and Lennard-Jones potentials), under the quasistatic limit, the evolution of low-frequency modes follows a distinct pattern: as the system approaches failure, a gradual decrease in frequency towards zero is anticipated [36, 70].

To see whether the normal modes of the Hessian could help to identify failure events, whenever there is a known avalanche

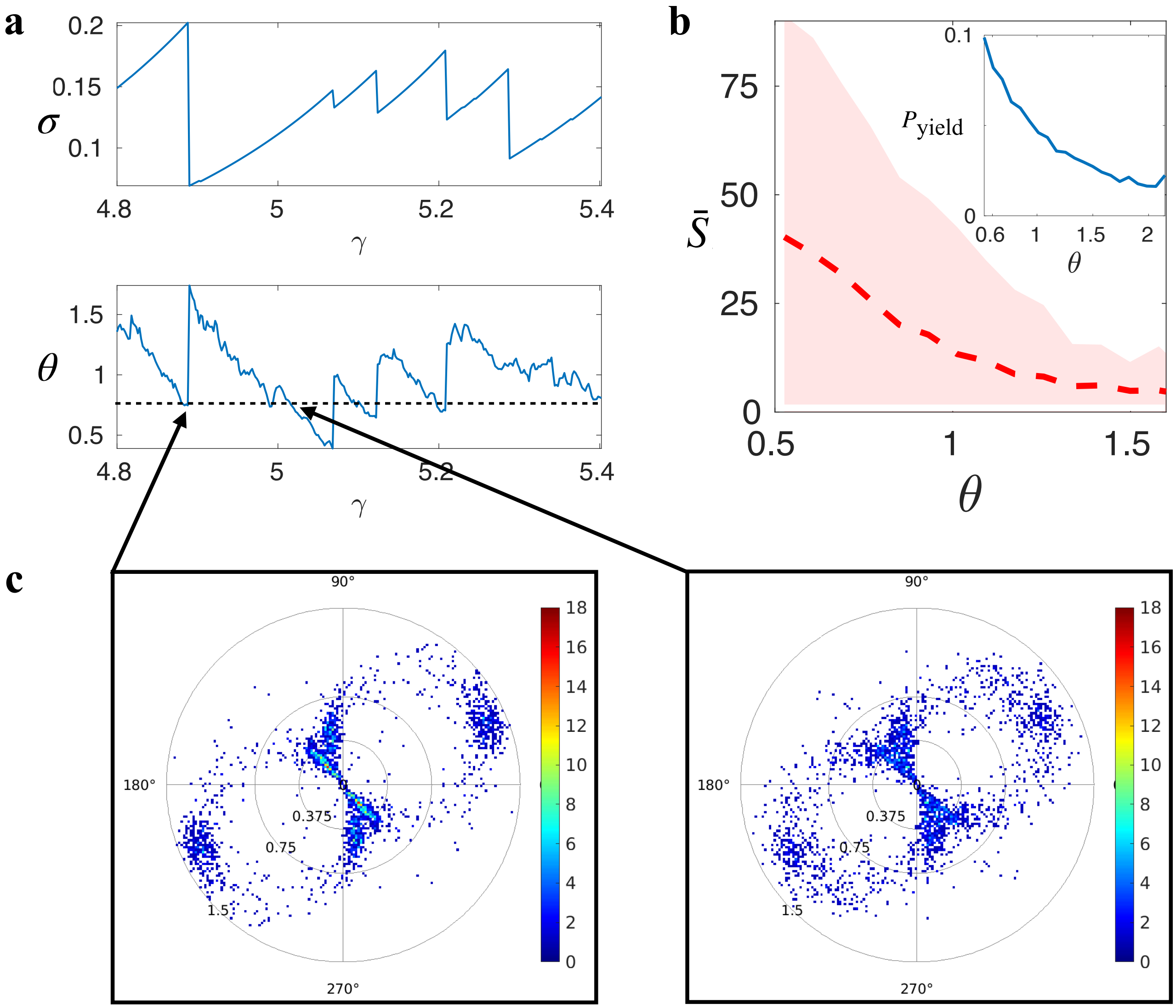

FIG. S1. A proposed exponent of instability. (**a**) An example of the evolution of tissue shear stress σ_{xy} and θ exponent as strain increases. (**b**) Dependence of average avalanche size and the exponent of instability θ. Inset: the probability to yield ρ_+ versus θ. (**c**) The polar distribution of edges vector in an unstable and stable configuration. Data shown in Figure 3 is extracted from a system with shape index $p_0 = 3.74$ in the solid regime.

in our simulation, we rerun the simulation starting at this particular strain but with the strain step decreased by 100 times and let the system approach to the avalanche again in the more detailed fashion. The low-frequency modes at the starting strain were then extracted from the Hessian. To keep track of the mode while the system evolves, we found the most similar mode to these starting low-frequency modes at each step and used them to represent the starting modes using the overlapping function $\Omega = \mathbf{e_i}.\mathbf{e_j}$, where $\mathbf{e_i}$ and $\mathbf{e_j}$ are eigenvectors of comparison. The overlap is shown on the right panel of Figure S2. In contrast to what was observed in other systems, we did not see a gradual decrease in the low-frequency mode eigenvalues. Instead, it is always a sharp decrease but not zero right at the onset of avalanches, no matter how detailed we zoom in on the approaching process. This non-smooth sudden drop in the eigenvalues at the onset of avalanches arises from the cuspiness of the energy landscape. Because of the cuspiness, there is no saddle point when the system approaches a rearrangement event and therefore the curvature is always positive.

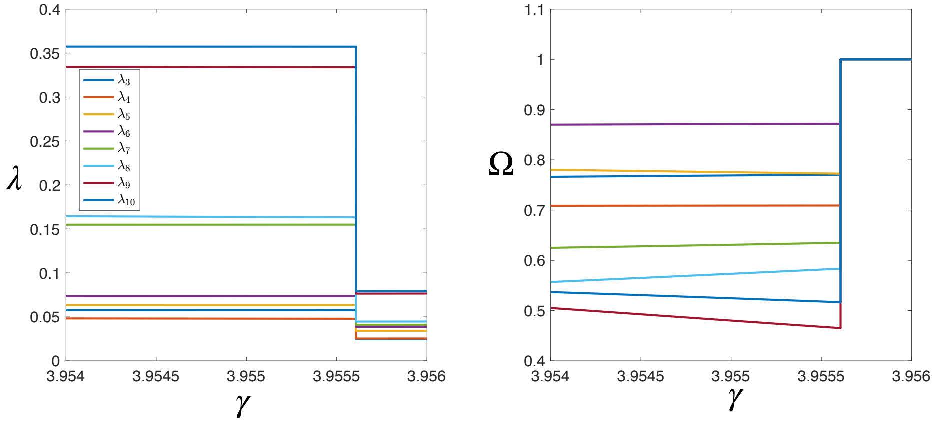

FIG. S2. Evolution of low-frequency mode near avalanches.

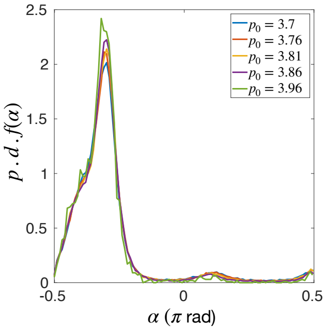

FIG. S3. The orientation distribution of T1-edges (edges undergo T1 transitions) at various values of p_0.

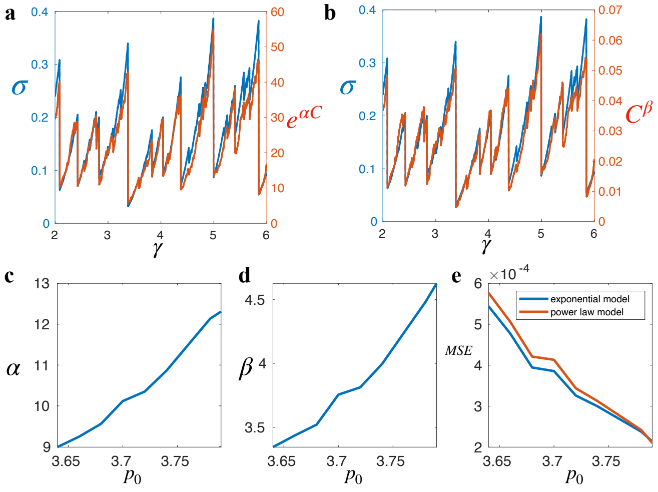

FIG. S4. Fitting σ_{xy} vs C^* to exponential and power-law model.

[1] A. Bonfanti, J. Duque, A. Kabla, and G. Charras, Fracture in living tissues, Trends in Cell Biology **32**, 537 (2022).

[2] E. Sadeghipour, M. A. Garcia, W. J. Nelson, and B. L. Pruitt,
